# Biochemical properties of L-arabinose isomerase from *Clostridium hylemonae* to produce D-tagatose as a functional sweetener

**DOI:** 10.1371/journal.pone.0196099

**Published:** 2018-04-23

**Authors:** Tien-Kieu Nguyen, Moon-Gi Hong, Pahn-Shick Chang, Byung-Hoo Lee, Sang-Ho Yoo

**Affiliations:** 1 Department of Food Science and Biotechnology, Sejong University, Seoul, Republic of Korea; 2 Carbohydrate Bioproduct Research Center, Sejong University, Seoul, Republic of Korea; 3 Department of Food Science and Biotechnology, College of BioNano Technology, Gachon University, Seongnam, Republic of Korea; 4 Department of Agricultural Biotechnology, Seoul National University, Seoul, Republic of Korea; 5 Center for Food and Bioconvergence, Seoul National University, Seoul, Republic of Korea; 6 Research Institute of Agriculture and Life Sciences, Seoul National University, Seoul, Republic of Korea; Universidade Nova de Lisboa, PORTUGAL

## Abstract

d-Tagatose has gained substantial interest due to its potential functionalities as a sucrose substitute. In this study, the gene *araA*, encoding l-arabinose isomerase (l-AI) from *Clostridium hylemonae* (DSM 15053), was cloned and expressed in *Escherichia coli* BL21 (DE3). This gene consists of 1,506 nucleotides and encodes a protein of 501 amino acid residues with a calculated molecular mass of 56,554 Da. Since l-AI was expressed as an intracellular inclusion body, this enzyme was solubilized with guanidine hydrochloride, refolded, and activated with a descending concentration gradient of urea. The purified enzyme exhibited the greatest activity at 50°C, pH 7–7.5, and required 1 mM of Mg^2+^ as a cofactor. Notably, the catalytic efficiency (3.69 mM^-1^sec^-1^) of l-AI from *C*. *hylemonae* on galactose was significantly greater than that of other previously reported enzymes. The bioconversion yield of d-tagatose using the *C*. *hylemonae* l-arabinose isomerase at 60°C reached approximately 46% from 10 mM of d-galactose after 2 h. From these results, it is suggested that the l-arabinose isomerase from *C*. *hylemonae* could be utilized as a potential enzyme for d-tagatose production due to its high conversion yield at an industrially competitive temperature.

## Introduction

d-Tagatose, a rare natural sweetener, is an isomer of aldohexose d-galactose and a C-4’-epimer (stereoisomer) of d-fructose. The taste of tagatose is closely equivalent to that of sucrose with no cooling effect or aftertaste, and has a relative sweetness value of 92% when compared to that of 10% solutions. This sugar has a significantly lower caloric value of 1.5 kcal/g, and the consumption of tagatose results in a reduced energy intake as well as promotion of weight loss at medically desirable rates [1, 2], which has also been used in the treatment of obesity [3, 4]. Owing to its lack of a glycemic effect [5], d-tagatose can be safely consumed by diabetic patients [6]. Several intensive studies have been conducted to investigate the different aspects of d-tagatose in food and pharmaceutical formulations, such as the stability of d-tagatose in food and drinks [7, 8], clinical trials for pharmaceutical applications [9], and consumer evaluations [10].

The production of d-tagatose from d-galactose can be achieved through chemical and enzymatic methods. However, the chemical method is limited for industrial applications because of the use of calcium as a catalyst, complicated processes, and the formation of chemical waste and unexpected by-products [11]. The production of d-tagatose by enzymatic methods is regarded as an environmentally friendly protocol, and has thus been studied more intensively in recent years. l-Arabinose isomerase (l-AI; EC 5.3.1.4) is the most effective biocatalyst for the isomerization of d-galactose to d-tagatose, which is an intracellular enzyme catalyzing the reversible isomerization of l- arabinose to l-ribulose and d-galactose to d-tagatose [12]. l-AIs originating from various microorganisms have been identified and biochemically characterized, including *Escherichia coli* [13, 14], *Lactobacillus plantarum* [15], *Bacillus subtilis* [16], *Lactobacillus reuteri* [17], *Bacillus licheniformis* [18], *Geobacillus thermodenitrificans* [11], *Thermoanaerobacterium saccharolyticum* [19], and *Thermotoga maritima* [20]. The ideal enzyme for production of d-tagatose in the food industry requires high thermostability and a weakly acidic pH optimum [5, 21]. The l-AI reaction at high temperature shifts the bioconversion equilibrium to d-tagatose production with an increasing reaction rate between d-galactose and d-tagatose, thereby decreasing the viscosity of the reaction mixtures and reducing the degree of microbial contamination [19, 22]. However, the thermophilic and hyperthermophilic l-AIs from various sources mainly present a slightly alkaline pH optimal range around 7–8.5 [18, 23], and show low activity at acidic pH conditions [24]. The mesophilic and thermophilic l-AIs also require Mn^2+^ and/or Co^2+^ as cofactors to enhance their isomerization activity and to maintain thermostability [23]. Moreover, ion-independent and Mn^2+^-dependent l-AIs are more favorable for the food industry owing to the toxicity of Co^2+^ [20, 23].

The objective of this study was to investigate a novel l-AI from *Clostridium hylemonae* DSM 1505, and its potential application in the environmentally friendly production of d-tagatose. The gene *araA* encoding l-AI from *C*. *hylemonae* DSM 15053 was cloned and expressed in *Escherichia coli* BL21 (DE3). The recombinant enzyme was purified and its biochemical characteristics were determined to optimize the production of d-tagatose from d-galactose by l-AI, as a highly valuable sweetener.

## Materials and methods

### Bacterial strains, plasmid, and chemicals

*C*. *hylemonae* (DSM 15053) was employed as a source of l-AI. *E*. *coli* JM 109 [F´ *traD36 proA*^+^*B*^+^ *lacI*^*q*^ *Δ(lacZ)M15/Δ(lac-proAB) glnV44 e14*^-^ *gyrA96 recA1 relA1 endA1 thi hsdR17*] (Promega, Madison, WI, USA) was applied as a host cell for gene cloning and DNA manipulation. *E*. *coli* BL21 (DE3) strain [*F*^-^ *ompT hsdS*_*B*_ (*r*_*B*_^-^ *mB*^+^*) gal dcm* (*DE3*)] (Novogen, Darmstadt, Germany) was applied as a host cell for expressing the enzyme. pGEM-T (Promega) and pET-28a(+) (Novogen, Darmstadt, Germany) vectors were employed as cloning and expression vectors, respectively. T4 DNA ligase was purchased from Elpis Biotech, Inc. (Daejeon, Korea). d-Tagatose and d-galactose of the highest purity were purchased from Sigma-Aldrich Co. (St. Louis, MO, USA). All other chemicals used in this study were of reagent grade.

### Cloning of the araA gene from *C*. *hylemonae*

*C*. *hylemonae* (DSM 15053) was cultured in brain heart infusion broth (BD, Sparks, MD, USA) under anaerobic conditions (Hungate tubes; N_2_) at 37°C for 24 h. The harvest was used directly as a template DNA for polymerase chain reaction (PCR). The full-length nucleotide sequence of the *araA* gene was obtained with two oligonucleotides designed as PCR primers, 5′-GAGACA*GGATCC*ATGATAAAAAGCAAAGAA-3′ (Forward) and 5′-ACATTC*CTCGAG*CTAAATCCCCAGCTTATAGGC-′ (Reverse), containing restriction sites of *Bam*HI and *Xho*I (underlined). The PCR was performed by iQ^™^5 Multicolor Real-Time PCR Detection Systems (Bio-Rad, Hercules, CA), and the amplified DNA fragment (1.5 kb) was ligated into the pGEM-T vector. The obtained *araA* sequence was compared with the original *araA* sequence in the National Center for Biotechnology Information (NCBI) database. The target gene, l-AI from *C*. *hylemonae* (*Ch*AI), was then subcloned into the expression vector pET-28a(+) with the *Nde*I and *Hind*III restriction sites. The sub-cloned pET28a-*Ch*AI was transformed into *E*. *coli* BL21 (DE3) for protein expression.

### Refolding and purification of the recombinant l-AI

After isopropyl-β-d-thiogalactoside induction at 16°C for 20 h, the cells were collected by centrifugation at 5,500 ×*g* at 4°C for 10 min, and the precipitant was resuspended in 20 mM Tris-HCl (pH 8.0). The suspended cells were disrupted by the Vibra^™^ Cell VC 750 disruptor (Sonics & Materials, Inc., Newtown, CT). The pellet harboring the inclusion body of l-AI was dispersed in a resuspending buffer [2 M urea, 20 mM Tris-HCl (pH 8.0), 0.5 M NaCl, and 2% Triton X-100)] to remove cell membrane proteins. After centrifugation, the pellet of recombinant l-AI as an inclusion body was solubilized in a binding buffer (pH 8.0) consisting of 20 mM Tris-HCl, 0.5 M NaCl, 6 M guanidine hydrochloride, and 2 mM β-mercaptoethanol. The dissolved inclusion body was collected by centrifugation at 13,000 × *g* at 4°C for 20 min, and the purification of 6× His-tagged recombinant l-AI was carried out by nickel-nitrilotriacetic acid (Ni-NTA) affinity chromatography (Qiagen, Hilden, Germany). The prepared enzyme solution was loaded into the column, and treateded with a washing buffer [20 mM Tris-HCl (pH 8.0), 0.5 M NaCl, 20 mM imidazole, 6 M urea, and 2 mM β-mercaptoethanol]. Refolding of the bound protein was conducted by applying a stepwise descending gradient with 6, 4, 2, and 0 M urea including 20 mM Tris-HCl (pH 8.0), 0.5 M NaCl, 20 mM imidazole, and 2 mM β-mercaptoethanol. The refolded enzyme was eluted initially with a refolding buffer [20 mM Tris-HCl (pH 8.0), 0.5 M NaCl, 20 mM imidazole, 20% glycerol, and 2 mM β-mercaptoethanol], and ended with elution buffer [20 mM Tris-HCl (pH 8.0), 0.5 M NaCl, 500 mM imidazole, and 2 mM β-mercaptoethanol]. The molecular mass of l-AI was estimated by 12% sodium dodecyl sulfate-polyacrylamide gel electrophoresis (SDS-PAGE) as introduced by Laemmli [25].

### Enzyme activity assay

The l-AI activity was measured according to the d-tagatose forming property using 10 mM of d-galactose, 25 mM of Tris-HCl buffer (pH 7.5), 1 mM of MnCl_2_, and 0.1 mg of the purified enzyme. The enzyme reaction was carried out at 35°C for 30 min, and was stopped by boiling for 10 min. After boiling, the mixture was cooled and centrifuged at 13,000 rpm for 5 min to remove the precipitant. The amount of d-tagatose produced was determined using high-performance liquid chromatography (HPLC) with a refractive index detector (RID) connected to a Sugar-Pak I analytical column (6.5 × 300 mm; Waters Corp., Milford, MA, USA). The column was eluted by de-ionized water at 78°C at a flow rate of 0.5 mL/min. One unit of l-AI activity was defined as the amount of enzyme producing 1 μmol of d-tagatose per minute at 35°C and pH 7.5. The concentration of protein was calculated following Bradford’s method using bovine serum albumin as the standard [26].

### Effect of metal ions on enzyme activity

The effects of metallic ions on l-AI activity were determined by testing various metal ions of MnCl_2_, CoCl_2_, ZnCl_2_, MgCl_2_, CuCl_2_, and CaCl_2_. The purified enzyme was treated with 10 mM of ethylenediaminetetraacetic acid at 4°C for 24 h to make the enzyme metal-free. The enzyme reactions were then carried out in the absence and presence of 1 mM divalent cations at 35°C for 30 min. The effect of the metal ion was determined using the same enzyme assay described above.

### Enzyme kinetics

The kinetic parameters of l-AI were determined using d-galactose as a substrate at eight different concentrations (30–600 mM) with the purified enzyme (0.1 mg) in 25 mM Tris-HCl buffer (pH 7.5) and 1 mM of Mg^2+^. The enzyme reactions were conducted at 50°C for 15 min. The amount of keto-sugar formed was determined by HPLC. Based on these reactions, the initial rates of the bioconversion of d-galactose were identified. The kinetic parameters (*K*_m_ and *k*_cat_) were investigated via nonlinear regression analysis with SigmaPlot 13.0 software (Systat Software Inc.; San Jose, CA, USA). All assays were carried out in duplicate.

### Enzymatic conversion of d-galactose to d-tagatose

The production of d-tagatose using 10 mM of d-galactose as a substrate by *Ch*AI was conducted in 25 mM Tris-HCl buffer (pH 7.5) with 1 mM Mg^2+^ and 1 mg/mL of purified *Ch*AI at 50–70°C for 10 h. Samples were drawn at the defined time and the amount of produced d-tagatose was measured by HPLC.

### Statistical analysis

All data are expressed as the mean ± standard deviation. Significant differences among the treatments were determined by one-way analysis of variance in IBM SPSS Statistics for Windows (version 21.0, IBM Corp., Armonk, NY). Statistical significance was indicated at a confidence level of 95%.

## Results

### Cloning and expression of the *Ch*AI gene

**Fig 1:**
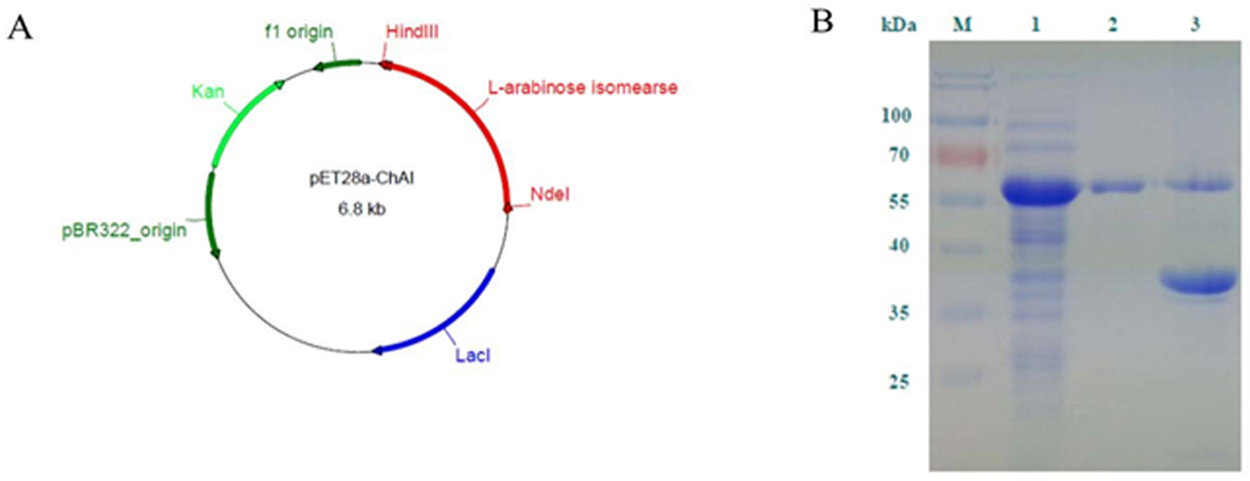
Construction of pET28a-*Ch*AI and SDS-PAGE analysis of *Ch*AI (B). (A) Map of the plasmid used for the expression of recombinant *Ch*AI in *E*. *coli* BL21 (DE3). (B) Lane M, protein size marker; lane 1, cell extract of *E*. *coli* BL21 (DE3) harboring 6× His-tagged *Ch*AI; lane 2, refolded 6× His-tagged *Ch*AI after purification using the Ni-NTA affinity column; lane 3, residual precipitate after solubilization of inclusion bodies in the buffer containing 6 M guanidine hydrochloride.

Based on the fully sequenced genomic DNA of *C*. *hylemonae* (NCBI accession number: NZ_GG657759.1), the *araA* gene encoding putative l-AI was successfully isolated and amplified by PCR. The amplified DNA fragment of about 1.5 kb was confirmed and compared with the original *araA* sequence in the NCBI database. The l-AI-encoding sequence was successfully constructed in the recombinant expression vector pET28a-*Ch*AI (Fig 1A). The recombinant l-AI expressed in *E*. *coli* formed an insoluble inclusion body, which was solubilized in guanidine HCl (6 M) resulting in ~72% dissolution of the aggregated recombinant l-AI (Table 1). The solubilized recombinant l-AI was refolded and purified through the Ni-NTA affinity chromatography column. Guanidine HCl-treated insoluble fraction of *Ch*AI showed two distinct protein bands on the SDS-PAGE gel, in which the molecular weights were estimated to be 57 kDa for fully denatured soluble *Ch*AI as shown in the lane 2 and 37 kDa for partially denatured enzyme (Fig 1B). Depending the denaturation conditions, it has been known that degree of protein denaturation might be different [27]. Incomplete denaturation by SDS treatment has also been reported for some thermostable enzymes [28]. The purified l-AI exhibited a specific activity of 0.44 U/mg at 35°C with d-galactose as a substrate.

**Table 1:** Purification of *6*× His-tagged recombinant *C*. *hylemonae* l-arabinose isomerase.

Fractions	Total protein (mg/ml)	Specific activity (U/mg)	Total activity (U)	Yield (%)
Total protein	232.59	-	-	-
Inclusion bodies	45.71	-	-	100
Solubilized protein	32.76	-	-	71.7
Ni-NTA	19.33	0.44	8.44	42.3

### Effect of temperature on enzyme activity and stability

**Fig 2:**
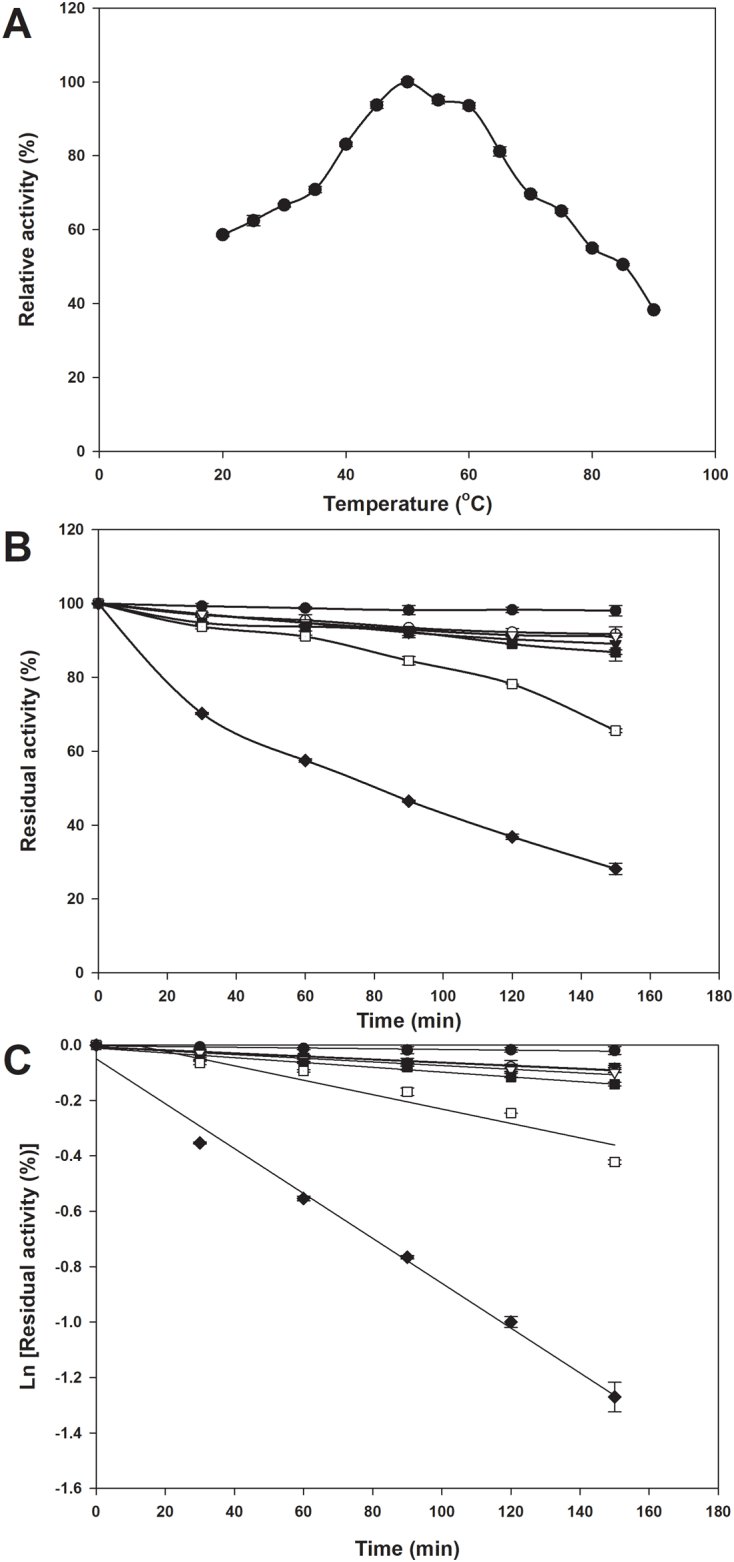
Effect of temperature on the activity and thermal stability of *Ch*AI. (A) The relative enzyme activity was assayed by d-tagatose formation for 30 min. Activity at the optimal temperature was defined as 100%. (B) Thermal stability profile of *Ch*AI at different temperatures: solid circles, 40°C; open circles, 45°C; solid triangles, 50°C; open triangles, 55°C; solid squares, 60°C; open squares, 65°C; solid diamonds, 70°C. The relative enzyme activity was assayed by tagatose production for 150 min. (C) The residual activities were measured at 40–70°C to determine the constant of enzymatic deactivation (*k*_*d*_). The initial activity was defined as 100%. The values are the means of two independent assays.

The temperature profile for *Ch*AI activity is shown in Fig 2. The optimal temperature of *Ch*AI was 50°C when d-galactose was used as a substrate (Fig 2A). The residual activity of *Ch*AI was approximately 81% and 38% at 65°C and at 90°C, respectively. The thermostability of *Ch*AI was investigated by measuring the enzyme activity over a temperature range from 40°C to 70°C for 150 min (Fig 2B). *Ch*AI displayed remarkable stability with 90% and 85% of the initial activity preserved at 45°C and 60°C, respectively. However, there was a considerable decrease in its activity along with the incubation time at over 65°C. The enzyme deactivation constant (*k*_*d*_) value for the thermal inactivation of *Ch*AI was calculated to be 0.486 s^-1^ and the half-life (*t*_*1/2*_) was 85.6 min at 70°C (Fig 2C).

### Effect of pH on enzyme activity and stability

**Fig 3:**
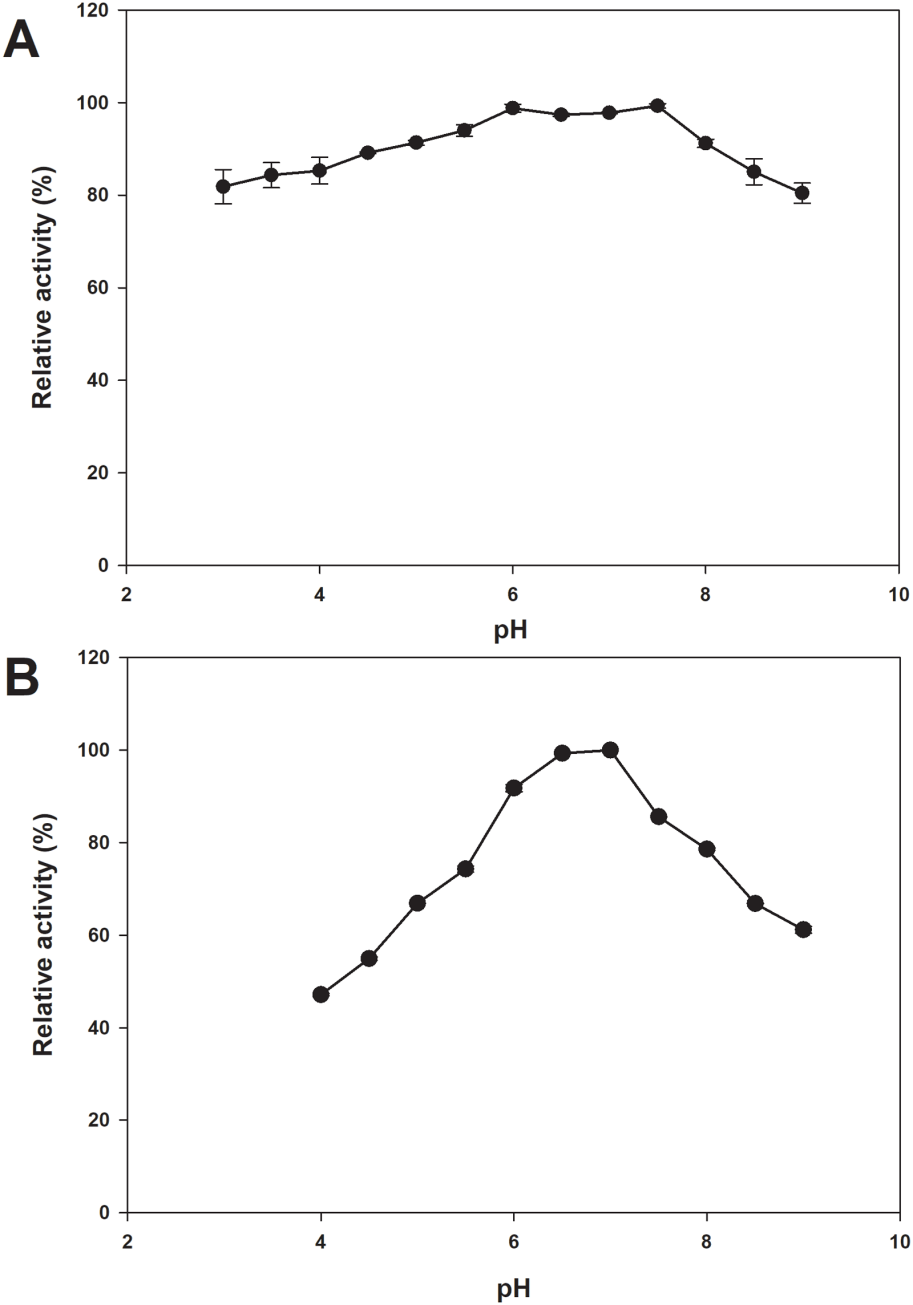
Effect of pH on the enzymatic activity and stability of *Ch*AI. (A) The enzyme activity was determined by monitoring the formation of tagatose from galactose at 35°C for 30 min incubation. (B) The enzyme stability of *Ch*AI was carried out at 4°C for 24 h. The remaining activity was determined in standard assay conditions.

The stable pH profile for the *Ch*AI activity of d-tagatose production exhibited quite a wide range, although the optimal pH was determined to be 7.5. Interestingly, *Ch*AI could maintain its high-level activity within the acidic pH range (3.0–5.5) as well as within the alkaline pH range (8.0–9.0), with over 80% of its maximum activity retained (Fig 3A). In addition, the pH stability profile for recombinant *Ch*AI showed that the enzyme was most stable at pH 6.5–7. It was also stable at a slightly acidic pH, with about 92% relative activity at pH 6.0 (Fig 3B).

### Effect of metal ions on enzyme activity

The *Ch*AI activity was measured with different types of divalent metal ions (1 mM), and no significant effect on tagatose production was detected by each of Ni^+^, Zn^2+^, Ca^2+^, and Cu^2+^ ions (Table 2). Several studies have reported that Cu^2+^ inhibits l-AI activity; [11, 18, 24, 29] however, this ion significantly enhanced the activity of *Ch*AI. In addition, the enzyme activity was increased by 20% of the relative activity compared to its initial activity in the presence of Mg^2+^. Moreover, 1 mM of Mg^2+^ was found to be the optimal concentration to most effectively enhance the activity of *Ch*AI within the concentration range tested of 0.2 to 2.0 mM (S1 Fig).

**Table 2:** Effect of various metal ions on *Ch*AI activity.

Metal ion (1 mM)	Relative activity^1^ (%)
Control^2^	100^b^ ± 2
EDTA	0^a^
Mn^2+^	111^b,c^ ± 2
Co^2+^	110^b,c^ ± 3
Mg^2+^	120^c^ ± 3
Zn^2+^	106^b^ ± 3
Ni^2+^	106^b^ ± 2
Ca^2+^	103^b^ ± 2
Cu^2+^	102^b^ ± 3

^1^ Values are the means ± standard deviations (*n* = 2).

Different superscript letters (a-c) associated with values in the same column indicate statistically significant differences (*P* < 0.05).

^2^ Native enzyme without EDTA treatment and extra metal ion.

### Kinetic parameters

*Ch*AI followed a Michaelis-Menten (MM) equation for d-tagatose production from d-galactose (S2 Fig). The apparent MM constant (*K*_m_) was 7.70 mM and the apparent catalytic constant (*k*_cat_) was 28.39 sec^-1^. The kinetics analysis showed that *Ch*AI exhibits higher substrate affinity toward d-galactose compared to l-AIs originating from other microorganisms (Table 3).

**Table 3:** Kinetic parameters of *Ch*AI reaction.

Microorganism	*K*_m_	*k*_cat_	*k*_cat_/*K*_m_	Reference
	(mM)	(s^-1^)	(s^-1^mM^-1^)	
*Clostridium hylemonae* 15053	7.70	28.39	3.69	This study
*Bacillus subtilis*	279	53.08	0.19	[36]
*Anoxybacillus flavithermus*	25.19	2.17	0.09	[37]
*B*. *stearothermophilus US100*	8.9	1.26	0.14	[22]
*G*. *stearothermophilus* T6	9.0	0.65	0.07	[28]
*Thermotoga maritima*	18.9	2.68	0.05	[20]
*Thermoanaerobacterium saccharolyticum* NTOU1	122	4.90	0.04	[19]
*Geobacillus thermodenitrificans*	408	3.40	0.008	[11]
*Thermotoga neapolitana*	250	13.50	0.054	[32]

### Conversion of d-galactose to d-tagatose using *Ch*AI

**Fig 4:**
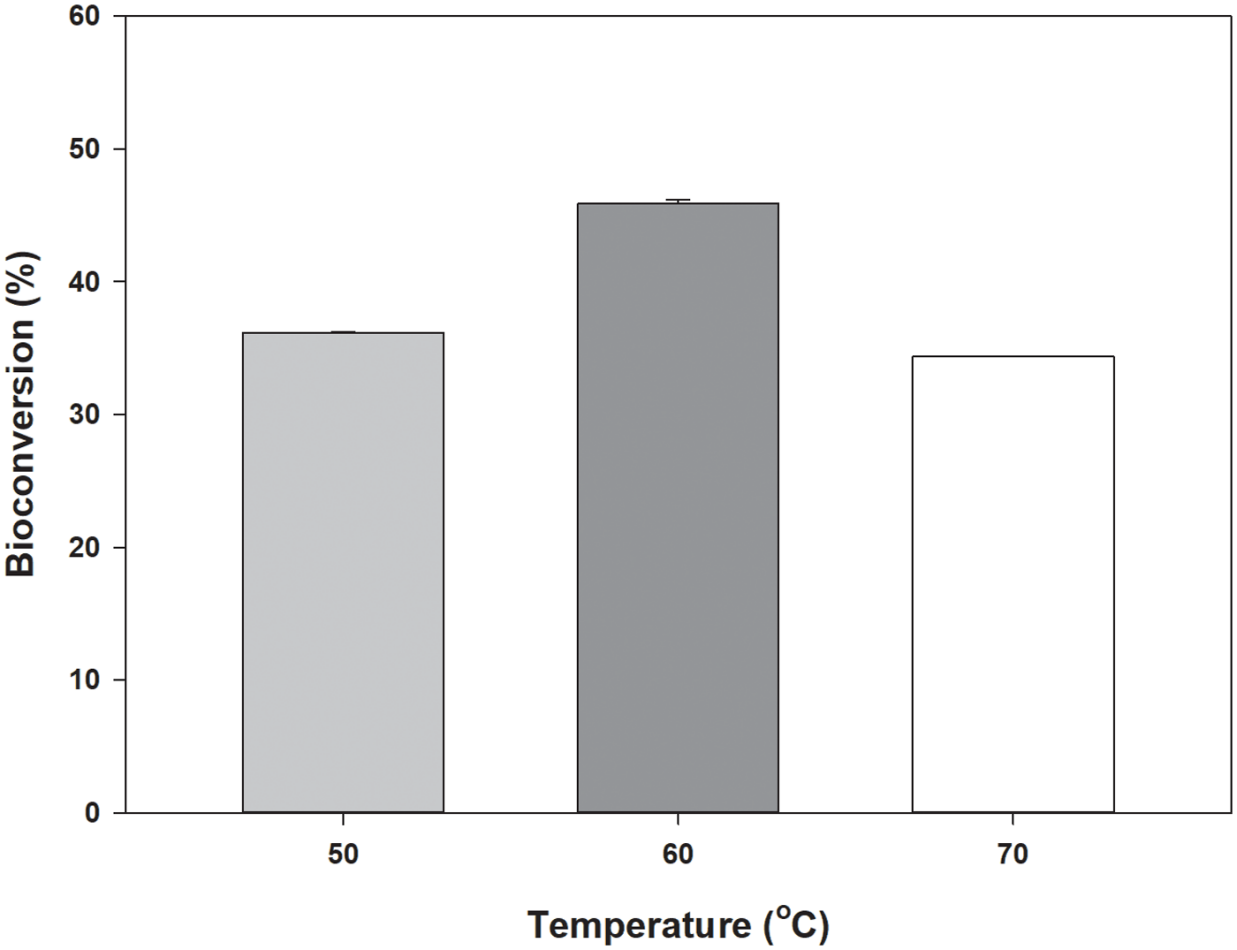
Temperature effect of the *Ch*AI reaction on the production yield of d-tagatose. Conversion yields of d-galactose to d-tagatose were carried out at a reaction temperature range between 50°C and 70°C for 10 h. The values are the means of two independent assays.

The bioconversion of d-galactose into d-tagatose using *Ch*AI was conducted at 50–70°C. The amount of d-tagatose reached equilibrium after 2-h incubation (S3 Fig). Interestingly, the production yields of d-tagatose increased from 36.1% to 45.9% as the reaction temperature increased from 50°C to 60°C, and then decreased to 34.4% with a further temperature increase to 70°C after 10 h of the reaction (Fig 4).

## Discussion

A novel l-AI from *C*. *hylemonae* was cloned, and its biochemical characteristics were determined focusing on high-value d-tagatose production by isomerization. The newly identified enzyme showed optimal activity at 50°C, pH 7.5, and required Mg^2+^ for enhancing its d-tagatose production property. Moreover, the enzyme displayed remarkable thermostability at 60°C and pH 6. d-Tagatose production by *Ch*AI resulted in a high conversion yield of approximately 46% at 60°C. For bio-industrial applications, d-tagatose production must be carried out at temperatures over 60°C, which can increase the bioconversion yield, reduce the viscosity of the reaction mixture, and decrease the possibility of microbial contamination [19, 20, 22, 30]. However, enzyme reactions above 70°C may result in undesirable reactions such as the formation of unexpected byproducts and a browning reaction [22]. Although *Ch*AI showed the greatest activity at 50°C, considerable levels of relative activity (93% at 60 min) and thermostability (85% after 150 min) remained at 60°C. Interestingly, the MM constant (*K*_m_) of *Ch*AI was lower and the turnover number (*k*_cat_) was higher than any other l-AIs reported previously (Table 3). The greatest catalytic efficacy (*k*_cat_/*K*_m_: 3.69 s^-1^mM^-1^) of *Ch*AI for tagatose production suggests that *Ch*AI would be a prospective candidate for industrial application at a competitive temperature and production rate.

Although the optimal pH of *Ch*AI activity for d-tagatose production was 7.0, it was still remarkably stable over a wide range of pH 3.0–10.0. The acid tolerance of l-AI is an important factor with respect to isomerization under acidic conditions to prevent the formation of inevitable byproducts and other environmental concerns [31]. l-AI from *C*. *hylemonae* maintained high activity and was sufficiently stable at pH 6.0 (~92% activity), which satisfy the industrial requirements of d-tagatose production as a green technology.

The d-tagatose production by *Ch*AI at 50°C was ca. 35%, which is almost identical to the previous result for a mesophilic l-AI. The reaction toward d-galactose is more dominant in an equilibrium reaction between d-galactose and d-tagatose from a mesophilic l-AI, resulting in a low conversion ratio of d-tagatose [11]. It was previously reported that the bioconversion equilibrium between d-galactose and d-tagatose shifted preferentially toward d-tagatose at higher temperature [20]. In particular, the conversion yield of d-galactose to d-tagatose using the l-AI from *E*. *coli* was less than 30% at 37°C [32, 33]. However, in the present study, the production yield of d-tagatose reached approximately 46% at 60°C. This specific enzyme displays the greatest conversion catalytic property as a mesophilic l-AI for d-tagatose production reported to date. *Thermotoga neapolitana* l-AI produced d-tagatose from 10 mM of d-galactose with yields of 68% at 80°C, while only 22% of d-tagatose was obtained at 50°C after 20 h [34]. It should be pointed out that within a relatively short reaction time of 6 h, this *Thermotoga* strain showed a bioconversion yield of 56% by the isomerization of d-galactose to d-tagatose at a very high temperature of 80°C [20]. Thus, hyperthermophilic l-AI produced d-tagatose from d-galactose effectively due to an equilibrium shift toward d-tagatose at high reaction temperature [11]. However, the browning reaction above 70°C will negatively affect the final products. Therefore, for commercial production, it may be preferable to utilize l-AI s that act well at around 60°C to limit undesirable color formation. It was previously reported that thermophilic l-AIs exhibited higher conversion yields than hyperthermophilic l-AI s at this temperature as well [34, 35]. Thus, considering the inherent biochemical properties, *C*. *hylemonae* l-AI could be applied as a potential enzyme for d-tagatose production by improving the protein expression systems (e.g., expression vectors or host) for commercial application.

## Supporting information

S1 Fig Effect of Mg^2+^ concentration on the activity of ʟ-AI from *C*. *hylemonae*. (TIF)

S2 Fig Lineweaver-Burk plot of *Ch*AI using ᴅ-galactose as substrate. The assays were conducted with the specified range of ᴅ-galactose concentration.
(TIF)

S3 Fig Effect of the reaction time of *Ch*AI on the production of ᴅ-tagatose at various temperatures: (*□*) 50°C; (*○*) 60°C; (△) 70°C. The values are the means of two independent assays.
(TIF)
