# Supplementary figures and images for: Biochemical properties of L-arabinose isomerase from Clostridium hylemonae to produce D-tagatose as a functional sweetener
Source: PLoS One. 2018 Apr 23;13(4):e0196099. doi: 10.1371/journal.pone.0196099 (PMC5912747; doi:10.1371/journal.pone.0196099)

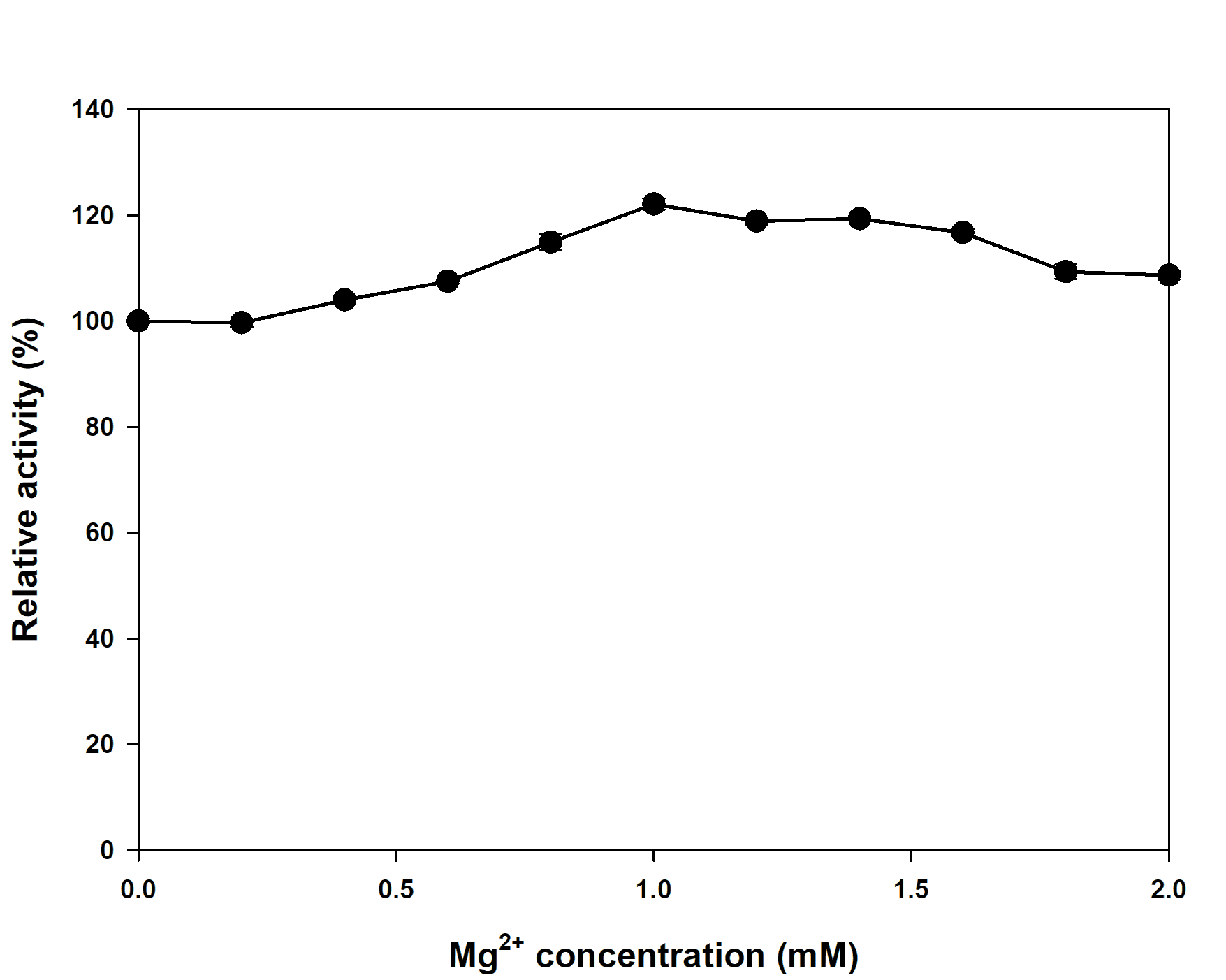

Supplement: S1 Fig — (TIF) [file pone.0196099.s001.TIF]

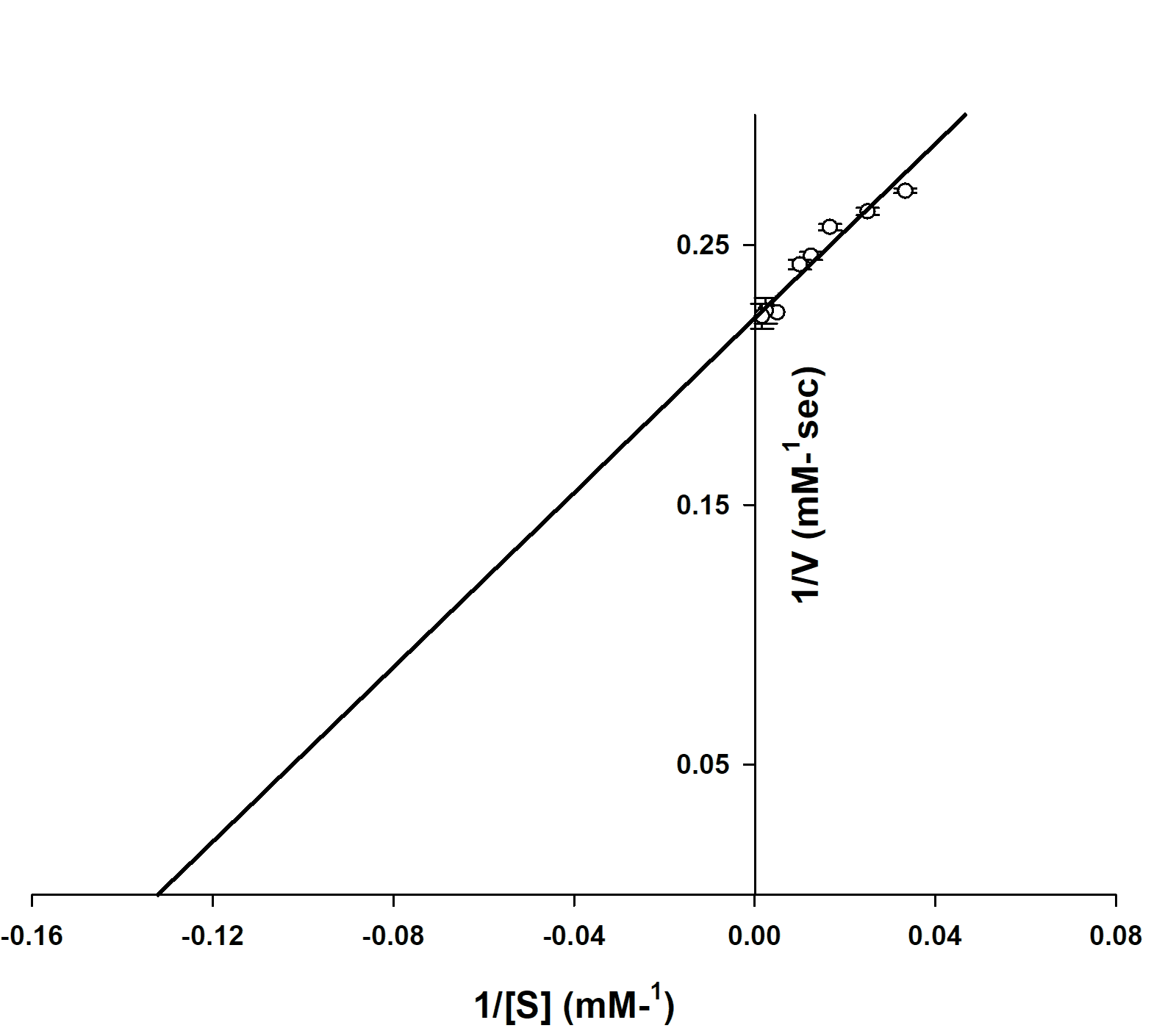

Supplement: S2 Fig — The assays were conducted with the specified range of ᴅ-galactose concentration. (TIF) [file pone.0196099.s002.TIF]

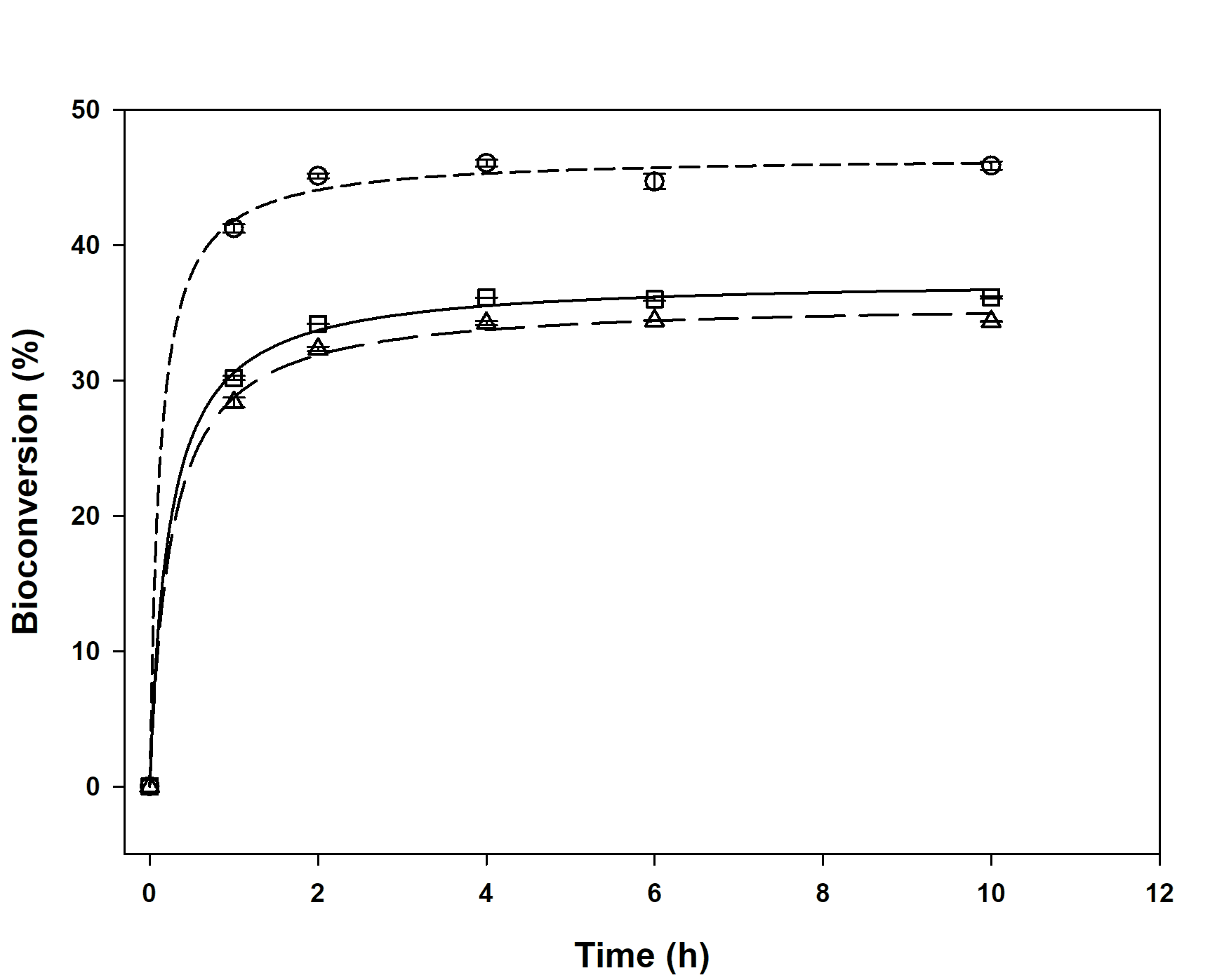

Supplement: S3 Fig — The values are the means of two independent assays. (TIF) [file pone.0196099.s003.TIF]
